# The Bicycle and Crime

**Published:** 1904-05

**Authors:** 


### THE BICYCLE AND CRIME.

The March number of the *Pall Mall Magazine* contains a characteristic article by Professor Lombroso on "The Bicycle and Crime." In reviewing this paper over the title, "To Perdition on a Wheel," the London *Hospital* says, Lombroso's work suffers from his devotion to one subject of study. When any new invention is introduced, he instinctively says, not "What will it do for mankind as a whole?" but "How will it affect the spread of crime?" Now, it would be difficult to name any addition to the comfort of the human race which has not an injurious effect on some person, and all that one can do is to take the balance of good and evil, and estimate the invention accordingly. This Professor Lombroso admits, but he declares that "no modern mechanism has assumed the extraordinary importance of the bicycle, either as a cause or an instrument of crime."

The importance of the bicycle as a cause of crime is easily disposed of. As a matter of fact, the bicycle has in itself nothing to do with it, and the same results would follow from the introduction of any novelty which most people would desire to possess. A young man wants a bicycle, but he can not afford to buy one, so he hires one for the day or week, and does not return it. This is the mere outcome of unregulated desire, uncontrolled by principle. A man of this type would just as readily steal a horse or a watch, or anything else that he happened to want as much as he did the bicycle. Blame the lack of honesty for the crime, not the poor, harmless bicycle. When the thief afterwards sells the stolen bicycle, as in some of the cases Lombroso quotes, we have only a vulgar case of obtaining goods under false pretenses; the bicycle is an accident in the affair, and assumes importance only because it is a chattel which can be so easily transported from one place to another. But the bicycle has never made a criminal of any man who might not under another temptation have lapsed into crime.

When it comes to the bicycle as an instrument of crime, the foundation of the accusation is more secure. The bicycle is swift and noiseless. The bicyclist can take side-roads where, even in populous countries, hardly any one will see him, and as there are, fortunately, many cyclists of most innocent intent, his passage, even if noted, will excite no comment. Even if he were known to be a thief, he can sweep away so fast that it would be difficult for any one to capture him. The bicycle is safer than the railway, for there are no fellow travelers to note one's appearance and fit their recollection into a published description of the criminal. Moreover, the railway leads only to more important centers, where the telegraph has probably carried the description of the person "wanted" by the police in advance of himself. But who could telegraph to all the villages, hamlets, and lonely cottages to which the cyclist may resort? We are here assuming that the criminal has committed his crime in a place where it would soon be discovered; but the ill-disposed cyclist has endless opportunities for crime on lonely roads and in isolated dwellings, on hapless women who can not make any effective struggle against his violence, nor find any one to whom they may accuse him before he is miles and miles away. Often, indeed, he may with impunity be guilty of offenses which those who suffer them shrink from publishing. In this way the bicycle certainly makes crime easy.

On the other hand, the bicycle has been used effectively in capturing criminals. If the offender has not too great a start, a good cyclist can follow on his tracks. A thief who tries to escape on foot has very little chance against a policeman mounted on a bicycle. It may come that a certain number of bicycles shall be regarded as part of the necessary equipment of every police station, as it is coming to be with postoffices, for the speedy conveyance of officials to wherever they are wanted. Professor Lombroso gives an illustration of a tandem cycle which is used in the State of Ohio for the conveyance of criminals. In this machine a policeman rides at each end, while the criminal, securely pinioned, sits in the middle. A machine like this, light, easily ridden, either with or without its freight of captured criminal, would be a convenient addition to the furniture of country police stations, and would prove both economical and useful.

Thus we find ourselves brought back to the legitimate uses of the bicycle, though perhaps not to the pleasantest of these. But the more one thinks of the matter, the more obvious it seems that the good the bicycle brings us outweighs the possible evils that may attend it. Every addition to human invention brings with it its own dangers. A hundred years ago there were no cases of gas poisoning, no murders in railway carriages, no boiler explosions at sea. Life was free from certain risks then, and a hundredfold more exposed to others equally serious. The bicycle is just like other things; it brings with it its dangers and its advantages likewise. Of the physical ills that may spring from over-indulgence in cycling, Professor Lombroso says nothing, though perhaps they are more real than those he deals with, and far more likely to affect average humanity. As far as it concerns man's body, Professor Lombroso has nothing but good to say of the cycle. He notes how cycling braces the muscles and steadies the nerves; he has himself seen a course of it cure the gravest forms of neurasthenia and melancholia. He has noted, too, in Italy, a result of the development of cycling which is noteworthy here also, and perhaps more necessary, how, namely, it has changed the character of roadside inns and refreshment houses. Formerly some form of alcohol was the only drink to be obtained in these. But your cyclist, though a thirsty soul, must needs be a temperate one. Drunkenness and cycling are incompatible. Therefore, cycling has brought into being a host of tea-houses, and largely increased the sale of all non-alcoholic drinks. The benefit of this to health and temperance can perhaps not be fully appreciated yet, but it is certainly making the generation which is now growing up far more abstemious than their fathers. And this of itself is a great good.

Thus we come back to the point that, though the bicycle will be to the criminally disposed a means of committing crime, it will be to the majority of humanity a means of healthy exercise, which will also teach much to eye, muscle and nerve. And as the majority of men are not criminals, we may rest satisfied that the good it brings will far outweigh the evil.—*The Dietetic and Hygienic Gazette.*

